# Osteocalcin Is Independently Associated with C-Reactive Protein during Lifestyle-Induced Weight Loss in Metabolic Syndrome

**DOI:** 10.3390/metabo11080526

**Published:** 2021-08-09

**Authors:** Silke Zimmermann, Maria Beatriz Walter Costa, Akash Mathew, Shruthi Krishnan, Jochen G. Schneider, Kirsten Roomp, Berend Isermann, Ronald Biemann

**Affiliations:** 1 Institute of Laboratory Medicine, Clinical Chemistry and Molecular Diagnostics, University of Leipzig, 04103 Leipzig, Germany; silke.zimmermann@medizin.uni-leipzig.de (S.Z.); Maria.WalterCosta@medizin.uni-leipzig.de (M.B.W.C.); akash.mathew@medizin.uni-leipzig.de (A.M.); shruthi.krishnan@medizin.uni-leipzig.de (S.K.); berend.isermann@medizin.uni-leipzig.de (B.I.); 2 Luxembourg Centre for Systems Biomedicine (LCSB), University of Luxembourg, Esch-sur-Alzette, 4362 Luxembourg, Luxembourg; josc012@outlook.com (J.G.S.); kirsten.rump@uni.lu (K.R.); 3 Department of Internal Medicine II, Saarland University Medical Center at Homburg/Saar, 66421 Homburg, Germany; 4 Centre Hospitalier Emile Mayrisch, Esch sur Alzette, 4240 Luxembourg, Luxembourg

**Keywords:** metabolic syndrome, osteocalcin, lifestyle-induced weight loss

## Abstract

Bone-derived osteocalcin has been suggested to be a metabolic regulator. To scrutinize the relation between osteocalcin and peripheral insulin sensitivity, we analyzed changes in serum osteocalcin relative to changes in insulin sensitivity, low-grade inflammation, and bone mineral density following lifestyle-induced weight loss in individuals with metabolic syndrome (MetS). Participants with MetS were randomized to a weight loss program or to a control group. Before and after the 6-month intervention period, clinical and laboratory parameters and serum osteocalcin levels were determined. Changes in body composition were analyzed by dual-energy X-ray absorptiometry (DXA). In participants of the intervention group, weight loss resulted in improved insulin sensitivity and amelioration of inflammation. Increased serum levels of osteocalcin correlated inversely with BMI (r = −0.63; *p* *<* 0.001), total fat mass (r = −0.58, *p* < 0.001), total lean mass (r = −0.45, *p* < 0.001), C-reactive protein (CRP) (r = −0.37; *p* < 0.01), insulin (r = −0.4; *p* < 0.001), leptin (r = −0.53; *p* < 0.001), triglycerides (r = −0.42; *p* < 0.001), and alanine aminotransferase (ALAT) (r = −0.52; *p* < 0.001). Regression analysis revealed that osteocalcin was independently associated with changes in CRP but not with changes in insulin concentration, fat mass, or bone mineral density, suggesting that weight loss-induced higher serum osteocalcin is primarily associated with reduced inflammation.

## 1. Introduction

The cluster of raised fasting plasma glucose, abdominal obesity, high triglycerides, and high blood pressure—all well-established cardiovascular risk factors—is commonly referred to as metabolic syndrome (MetS). Its increasing prevalence represents a major public health burden, as individuals with MetS have twice the risk of developing cardiovascular disease (CVD) and a five times elevated risk for type 2 diabetes mellitus (T2DM) [1]. In MetS, lifestyle-induced weight loss is regarded an effective therapy to reverse insulin resistance, to prevent T2DM, and to reduce low-grade inflammation and CVD [2,3]. 

A link between (I) bone and (II) energy metabolism and glucose homeostasis has been proposed [4,5,6]. Of the different bone turnover markers, reduced osteocalcin levels are associated with overweight and MetS parameters that include higher waist circumference, higher triglyceride and glucose levels, increased blood pressure, and lower HDL-cholesterol [7,8,9]. Of note, in one study, osteocalcin levels were increased following 12 weeks of exercise training and correlations between changes in BMI, HOMA, adipose tissue-derived adiponectin, and osteocalcin were observed [10].

Osteocalcin is a noncollagenous protein secreted by osteoblasts, reflecting osteoblast activity [11]. Interestingly, osteocalcin knockout mice are characterized by an increase in visceral fat mass, as well as hyperglycemia, hypoinsulinemia, and reduced β-cell mass [12], indicating that osteocalcin regulates glucose metabolism and insulin secretion in mice [13,14,15]. Furthermore, administration of osteocalcin was shown to improve insulin sensitivity and to decrease the severity of obesity and T2DM in mice fed with a high-fat diet [14]. A relationship between reduced osteocalcin levels and obesity was also identified in apparently healthy children, independent of their pubertal development [16]. In addition to its effects on insulin, osteocalcin has also been shown to suppress proinflammatory cytokine secretion, while stimulating the anti-inflammatory interleukin 10 in whole organ adipose tissue culture [17]. However, the relationship between changes in osteocalcin, body composition, metabolic parameters, and systemic low-grade inflammation following lifestyle-induced weight loss in MetS remains unknown. To address this question, we determined the changes and interrelations of osteocalcin with clinical, metabolic, and inflammatory parameters following lifestyle-induced weight loss in 74 well-defined individuals with MetS in a prospective study.

## 2. Results

### 2.1. Clinical and Laboratory Parameters

**Figure 1:**
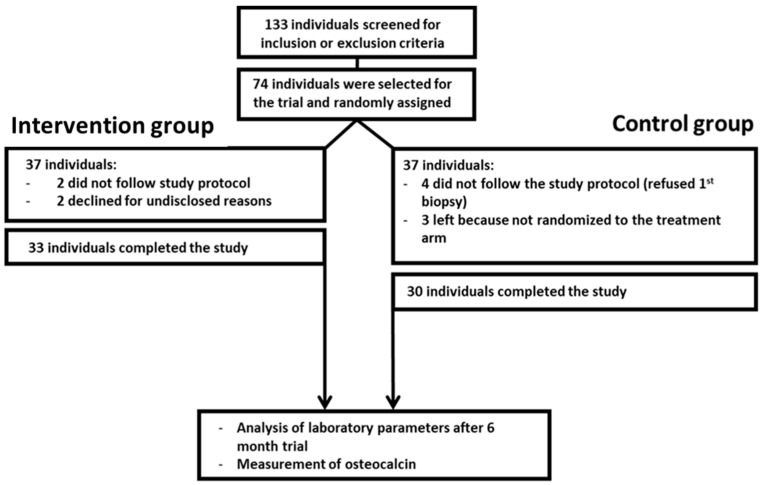
Schematic of study design. The study was embedded within a two-armed, controlled, monocentric, randomized, 6-month intervention period. Paired blood samples were collected before and after the 6-month intervention period. Thirty participants in the control group and 33 in the intervention group completed the study and were included in the data analysis. Two participants of the intervention group and four of the control group did not follow the study protocol, and three participants of the control group left the study due to not being included in the weight loss program (intervention group). The remaining two dropouts of the intervention group declined to continue for undisclosed reasons.

**Figure 2:**
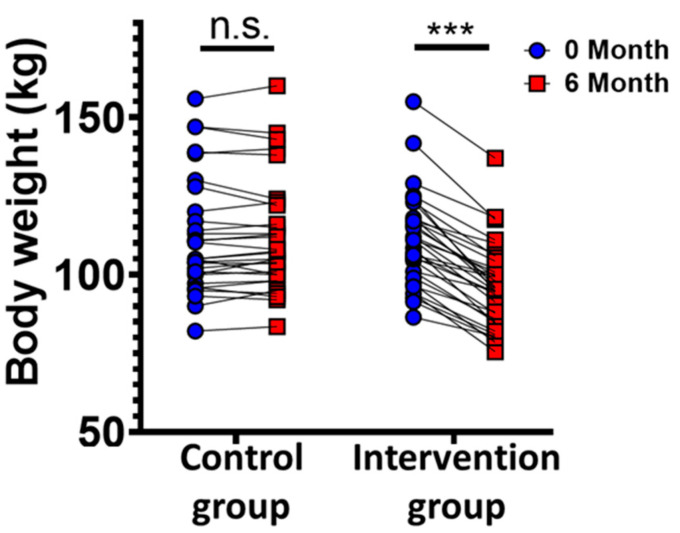
Line graph of total weight loss in both groups. Data is presented as total body weight in kg. The Wilcoxon signed-rank test was used to analyze differences between paired samples with *n*  =  33 intervention, *n*  =  30 control, *** *p*  *<*  0.001.

**Figure 3:**
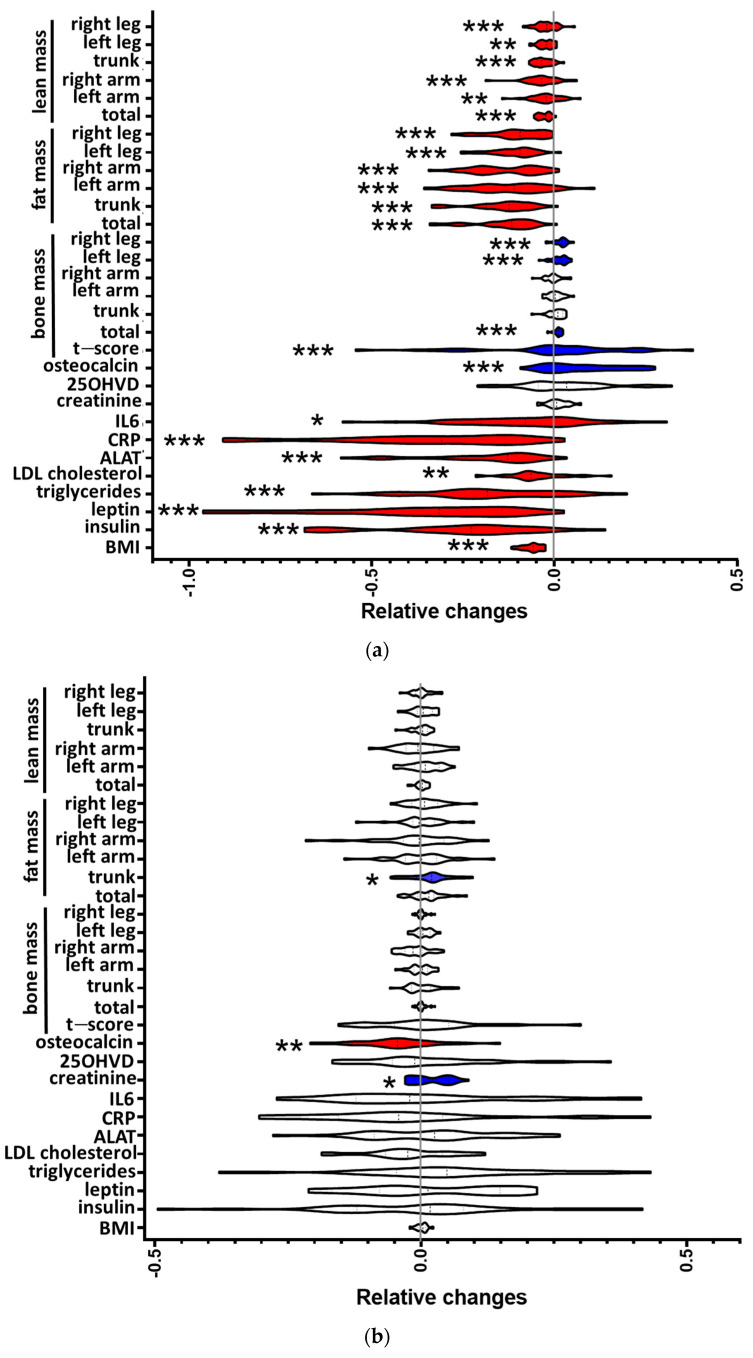
(**a**) Changes in body composition and clinical and laboratory parameters in participants of the intervention group. Violin plots of log ratios of given parameters before and after the 6-month intervention period. The Wilcoxon signed-rank test was used to analyze differences between paired samples; * *p*  *<*  0.05; ** *p*  *<*  0.01; *** *p*  *<*  0.001. The grey line denotes baseline levels. The red color indicates a significant reduction while the blue color reflects a significant increase in the indicated parameter after the intervention period. (**b**) Changes in body composition and clinical and laboratory parameters in participants of the control group. Violin plots of log ratios of given parameters before and after the 6-month study period. The Wilcoxon signed-rank test was used to analyze differences between paired samples; * *p*  *<*  0.05; ** *p*  *<*  0.01. The grey line denotes baseline levels. The red color indicates a significant reduction while the blue color reflects a significant increase in the indicated parameter after the study period.

We analyzed serum osteocalcin levels before and after lifestyle-induced weight loss in individuals with metabolic syndrome (MetS). Seventy-four nonsmoking men (45–55 years old) with MetS were randomized to a lifestyle-induced weight loss program (supervised via telemonitoring) or to a control group. Clinical and laboratory parameters and osteocalcin concentrations were determined in fasting blood samples before and after the six-month weight loss intervention (Figure 1, Supplementary Table S1). Thirty participants in the control and 33 participants in the intervention group completed the study and were included in the data analysis. Two participants of the intervention group and four of the control group did not follow the study protocol, and three participants of the control group left the study because they were not selected for the intervention group. The remaining two dropouts of the intervention group declined to continue for undisclosed reasons. The study populations did not differ in regard to the distribution of age, sex, or MetS parameters. Participants of the intervention arm reduced their individual body weight by at least 5%. Notably, 76% reduced their body weight by at least 10%, and 40% reduced their body weight by at least 15%, similar to previous reports [18] (Figure 2). To address the question of whether weight loss affects osteocalcin levels in individuals with MetS, and whether osteocalcin is associated with metabolic parameters, body composition, or inflammation, we determined the changes and interrelations of predefined parameters before and after lifestyle-induced weight loss (Figure 3, Supplementary Table S1). In participants of the intervention group, lifestyle-induced weight loss resulted in reduced levels of insulin, leptin, LDL cholesterol, triglycerides, C-reactive protein (CRP), and alanine aminotransferase (ALAT) (Figure 3a).

Reduction in body weight was mainly attributable to a significant reduction in individual body fat mass, as measured by DXA (−23,57%, *p* < 0.001; Figure 3a). In controls, BMI and other parameters of body composition remained unchanged, and trunk fat mass even increased by 4.78% [18] (Figure 3b). In both groups, no progression to overt type 2 diabetes was observed. We observed an increase (*p* = 0.01) in bone-derived osteocalcin levels in participants of the intervention group following the 6-month weight loss trial (Figure 3a). Meanwhile, in participants of the control group (Figure 3b), we observed a slight decrease in osteocalcin after the 6-month trial period. DXA was performed at baseline and after the 6-month trial period in order to evaluate lifestyle-induced changes in body composition. Participants in the intervention group showed an increased t-score and increased total bone mass, mainly due to an increase in bone mass in the legs (Figure 3a). In addition, we observed a reduction in body fat mass in all analyzed regions (total, trunk, both arms, both legs) in the intervention group. Similarly, lean mass was reduced in all measured body regions after weight loss. In the control group, a slight increase in trunk fat mass, increased creatinine levels (Figure 3b), and decreased GFR levels (Supplementary Table S1) were observed in addition to the decreased osteocalcin, while other parameters remained unchanged (Figure 3b).

### 2.2. Correlation Analysis of Osteocalcin and Parameters of the Metabolic Syndrome

**Figure 4:**
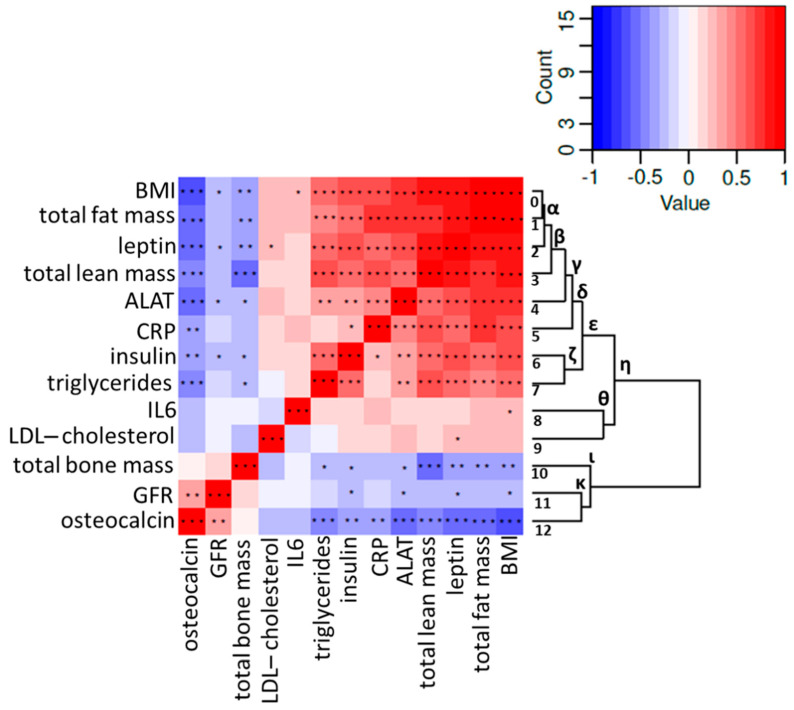
Correlation between OC, metabolic markers, body composition, and inflammation during the 6-month study in both groups. The heatmap (**left**) represents Spearman correlations between relative changes in variables. Colors indicate the correlation coefficient, ranging from blue (−1,0) to white (0,0) to red (1,0). Stars represent significant *p*-values: * *p* < 0.05; ** *p* < 0.01; *** *p* < 0.001. The dendrogram (**right**) indicates the hierarchical relationship between variables. Variables with similar patterns of correlation are clustered together. Numbers indicate chunks, Greek letters indicate clades.

We calculated the correlation between changes in osteocalcin, metabolic markers, body composition, and inflammation. We found correlations of the relative (log ratios) changes in osteocalcin with BMI (r = −0.63; *p* < 0.001), leptin (r = −0.53; *p* < 0.001), ALAT (r = −0.52; *p* < 0.001), CRP (r = −0.37; *p* < 0.01), insulin (r = −0.4; *p* < 0.001), and triglycerides (r = −0.42; *p* < 0.001) (Figure 4). Hence, an increase in osteocalcin was found to be associated with lifestyle-induced weight loss and the associated metabolic and inflammatory improvements. Interestingly, while a correlation was found between osteocalcin and total fat mass (r = −0.58; *p* < 0.001) and between osteocalcin and total lean mass (r = −0.45; *p* < 0.001), we found no correlation between osteocalcin and total bone mass (r = 0.08).

The dendrogram (Figure 4, Supplementary Figure S1) shows that osteocalcin connects closely to bone mass changes (clade ι). GFR and osteocalcin (chunk 11 and 12) join together first in the branching diagram and are closely connected to changes in bone mass (chunk 10). Of note, this clade (clade ι) is clearly separated from the others. Body composition parameters BMI, total fat mass, leptin, and total lean mass are linked (clades α, β) and more distinctly related to changes in inflammatory and liver-injury markers CRP and ALAT, respectively (clade δ). Insulin level changes and triglyceride level changes were strongly similar (clade ζ), and on a higher level connected to all previously mentioned parameter changes in inflammation and body composition (clade ε) (Figure 4).

### 2.3. Multiple Regression Analysis between Osteocalcin and Parameters of the Metabolic Syndrome

**Figure 5:**
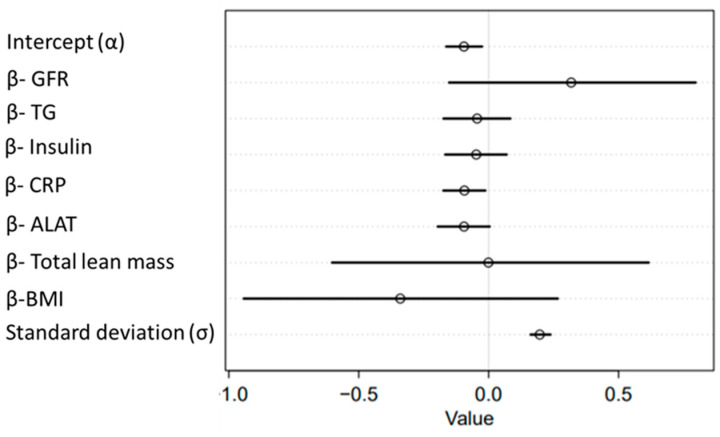
Multiple linear regression analysis between osteocalcin (dependent) and metabolic, inflammatory, and body composition variables (independent). *X*-axis, confidence intervals (CIs) of the strength of correlation to osteocalcin change; *y*-axis, parameters of the regression: slope coefficients of each variable (beta), intercept (alpha), and standard deviation (sigma). Considering all slope coefficients, beta-CRP is the only parameter with a completely negative CI, indicating that CRP level change is associated with OC level change in lifestyle-induced weight loss. All other slope CIs range from negative to positive values, indicating that their variables alone cannot predict OC level change. TG, triglycerides; CRP, C-reactive protein; ALAT, alanine aminotransferase; BMI, body mass index; GFR, glomerular filtration rate.

Considering all variables that were significantly correlated with osteocalcin (Figure 4: BMI, total fat mass, leptin, total lean mass, ALAT, CRP, insulin, GFR, and triglycerides) as potential independent determinant variables, we performed a Bayesian multiple linear regression analysis to identify which of these variables are predictive for changes in osteocalcin (Figure 5). To avoid multicollinearity in the regression, we excluded leptin from the independent variables, since it was highly associated with insulin and glucose (not shown), as well as ‘total fat mass’, due to it being highly associated with BMI (not shown). The regression analysis was performed with data of both groups. In summary, we fit the regression model to the data and obtained a posterior distribution that describes the relation between the independent and dependent variables. The regression showed that CRP level change is the most promising variable to predict osteocalcin level change in lifestyle-induced weight loss. At a confidence interval (CI) of 90%, the probability mass of beta-CRP does not contain zero, while the other variables do (mean beta-CRP = −0.09, with a standard deviation of 0.05, ranging from −0.18 to −0.01 with a 90% CI, Figure 5).

## 3. Discussion

To examine the effect of weight loss-induced changes in body composition and metabolic parameters on osteocalcin levels, we determined osteocalcin serum levels, body composition, and metabolic parameters in a prospective, blinded, randomized weight loss study. Multiple linear regression analysis revealed that changes in insulin, leptin, bone mass, and adipose tissue did not show relevant associations with increased serum osteocalcin levels following weight loss. The only association we found to be independently related to osteocalcin was that of C-reactive protein (CRP). This raises the question as to whether the previously reported associations of osteocalcin with metabolic parameters are, in fact, indirect, instead reflecting the subchronic inflammatory state associated with MetS.

The correlation between osteocalcin and inflammation in our study is in agreement with observations in nontraumatic fractures in which osteocalcin was inversely associated with CRP, independently of metabolic, cardiovascular, or chronic diseases [19,20]. Congruently, Riquelme-Gallego et al. identified an association between reduced osteocalcin levels and increased cardiovascular risk in MetS patients without diabetes mellitus type 2 [21]. Furthermore, osteocalcin has been associated with IL6 and CRP in T2DM [22,23]. Likewise, we observed a decrease in IL6; yet this association did not reach significance, possibly due to the low number of participants that were enrolled in our study. In support of a close interaction between osteocalcin and CRP, osteocalcin has been shown to suppress proinflammatory cytokine secretion (TNF-alpha and IL6), while stimulating anti-inflammatory interleukin−10 in vitro [17]. The latter finding raises the intriguing possibility that osteocalcin is not associated with, but actually modulates, low-grade inflammation in the setting of MetS. As our study was not designed to determine causality, future studies are needed to address this possibility.

The observed increase in circulating osteocalcin levels following lifestyle-induced weight loss is congruent with cross-sectional observations showing reduced osteocalcin in patients with obesity or MetS [24,25,26,27].

Given the predominant production and secretion of osteocalcin by osteoblasts, we initially expected that an increase in osteocalcin would be related to changes in bone mass. Contrary to our assumption, however, osteocalcin was not correlated with changes in bone mass. Instead, it was correlated with reduced adipose tissue mass, reduced lean mass, and changes in metabolic and inflammatory parameters that include fasting insulin levels, leptin, triglycerides, CRP, and ALAT. However, regression analysis revealed that the only parameter independently associated with osteocalcin was CRP. Of note, vitamin D and glomerular filtration rate did not change following lifestyle-induced weight loss, excluding the hypothesis that observed changes in osteocalcin and bone mass were influenced by kidney function or vitamin D deficiency in our study [28,29]. Taken together, these findings imply that increased osteocalcin levels following lifestyle-induced weight loss are primarily associated with reduced low-grade inflammation, while improved peripheral insulin sensitivity and liver parameters may be secondary to these changes.

Strengths of the current study include a well-characterized study population with no differences at baseline, a prospective study design, and a high compliance of participants as a result of daily telemonitoring and weekly letters commenting on individual weight progress. All laboratory measurements were performed following standard operating protocols and laboratory technicians were blinded. We used dual-energy x-ray absorptiometry, which is the standard method of measuring bone mineral density in clinical and research settings, to analyze body composition before and after lifestyle-induced weight loss. In order to generate a homogenous study group, only middle-aged Caucasian males with MetS were included in this study. While this increased homogeneity among participants, the results therefore cannot be generalized to other ethnical groups, genders, or individuals without MetS. An additional limitation is the relatively small sample size. The observational period was 6 months, precluding conclusions on the long-term effects of weight loss on osteocalcin. In the controls, we observed decreased osteocalcin levels following the 6-month study period. As the study started in May and ended in November, a possible reason for reduced osteocalcin levels in the control group are seasonal effects. Assuming a comparable seasonal effect in the intervention group, correction of serum osteocalcin levels for a potential seasonal effect in the weight loss group would potentially result in even higher serum osteocalcin levels. As we did not observe changes in vitamin D levels, vitamin D-driven seasonal effects on bone metabolism and osteocalcin can be excluded. In addition, our study does not allow us to deduce any causal relationship between osteocalcin and CRP. Further research is necessary to study the mechanistic interaction between osteocalcin and CRP and, potentially, other inflammatory markers.

In summary, 6 months of controlled lifestyle-induced weight loss led to an increase in bone-derived osteocalcin levels, which are associated with reduced inflammation, represented by CRP measurements, in MetS. Interestingly, the increased osteocalcin serum levels were not correlated to changes in bone mineral density. Since inflammation and insulin sensitivity are linked, our study suggests that osteocalcin reflects changes in insulin sensitivity only indirectly, presumably due to concomitantly improved inflammation.

## 4. Materials and Methods

### 4.1. Research Design

The study was embedded in a prospective, two-armed, controlled, monocentric, randomized, 6-month intervention trial to identify changes in clinical and laboratory parameters in individuals with MetS following lifestyle-induced weight loss. For this purpose, paired blood samples and subcutaneous adipose tissue biopsies were collected at the Institute of Clinical Chemistry and Pathobiochemistry, Otto von Guericke University, Magdeburg, Germany. The trial was registered at the German Clinical Trials Register (ICTRP Trial Number: U1111-1158-3672) in July 2014. The trial included nonsmoking, nondiabetic men aged between 45 and 55 years with MetS, as defined by the consensus definition in 2009 [1]. Three out of five criteria needed to be met: abdominal obesity (waist circumference > 102 cm or BMI > 30 kg/m^2^); fasting triglyceride concentration ≥ 1.7 mmol/L (or pharmaceutical intervention); high-density lipoprotein (HDL) cholesterol *<* 1.00 mmol/L; fasting glucose ≥ 5.6 mmol/L (or pharmaceutical intervention); and blood pressure ≥ 130/85 mmHg or treatment for hypertension. Exclusion criteria were smoking, diabetes mellitus type 2, surgical procedure for weight loss within the previous 6 months, severe renal dysfunction (creatinine concentration > 2.0 mg/dl), active liver disease, obesity of known endocrine origin, or inability to walk at least 30 min per day. Participants were recruited by an advertisement in a regional newspaper. Out of 133 individuals screened for inclusion or exclusion criteria from May 2012 to August 2012, 74 individuals were selected for the trial. All participants of the study were instructed to reduce calorie intake and maintain a low-carbohydrate diet with preference for low-glycemic index carbohydrates via structured education. The subjects were advised to increase their usual daily physical activity, such as walking or cycling, rather than to engage in particular sports. The recommendation was to perform these activities moderately but steadily, slowly enough to be able to talk at the same time, and to keep the pulse below 120/min. The key difference between the two groups was a daily telemetric report by the study participant and weekly written feedback from a doctor in the intervention arm, while the control group was only instructed once at the beginning of the study [30]. Beyond these instructions, no special diet or recommendations, e.g., for physical activities, were given. Individuals were randomly assigned to a 6-month lasting telemonitored lifestyle-induced weight loss program or a control arm by a web-based randomization tool using permuted block randomization with stratification on BMI (Randomization in Treatment Arms (RITA); University of Lübeck, Lübeck, Germany). A blinded investigator, who did not have any interaction with the participants during the screening and enrolment process, managed group allocation assignments. Participants of both arms were linked to an identification number and samples were collected in sequentially numbered containers. Hence, laboratory technicians were blinded regarding the origin and allocation of the samples. Participants of the intervention arm received accelerometers measuring their daily activity and instructions for daily data transmission of body weight, physical activity, and approximate caloric intake [30]. Participants of the intervention arm received regular feedback by weekly letters commenting on their individual weight progress and daily exercise-related energy expenditure. At baseline and after 6 months, subcutaneous adipose tissue biopsies and peripheral blood samples were obtained from participants of both arms. All study participants were examined after 3 months and screened for fasting blood glucose and glycated hemoglobin (HbA1c) to prevent complications secondary to progression to overt type 2 diabetes. The expression profile of microRNAs in paired blood and subcutaneous adipose tissue will be analyzed as a primary outcome (not part of this report).

### 4.2. Dual Energy X-ray Absorptiometry

We applied the DXA method (dual-energy X-ray absorptiometry) to determination of the bone density on the femoral neck. The relevant measurement region (ROI, region of interest) was determined and the measured radiation absorption in grams per square centimeter (g/cm^2^) was calculated according to an area measurement. When measuring the spine, L1–L4 are used as standard. Densitometric measurement results of an individual are compared with an age- and gender-specific control group. A normative database, as implemented by the device manufacturers in the operating software, is indispensable for the interpretation of patient results. The measured bone mineral density is given as a t value (t-score). The t-score refers to the peak bone mass divided by the standard deviation of this mean. The value is given as a unit in standard deviations. Evaluation of the bone density should only be carried out primarily on the femoral neck. DXA requires low radiation exposure, easy standardization, and availability of the method. The t-score is defined as the difference between a measured bone mineral density (BMD) and the expected normal value divided by the population standard deviation.

### 4.3. Clinical and Laboratory Parameters

Osteocalcin levels were measured at baseline and at the end of the study (after 6 months) in paired serum samples using a commercially available sandwich enzyme-linked immunosorbent assay (Human osteocalcin ELISA, Cat. No. RIS002, BioVendor Research and Diagnostic Products GmbH) at the Institute for Clinical Chemistry and Pathobiochemistry, Otto von Guericke University, Magdeburg, Germany. The intra-assay coefficients of variation (CVs) ranged from 3.1% to 4.7%, the interassay CVs ranged from 3.5% to 5.6%, and the lower limit of detection was 0.1 ng/mL, as according to the manufacturer. Body weight, height, waist circumference, and blood pressure were measured by qualified medical personnel according to standard operating protocols at baseline and after 6 months. Body composition was analyzed by dual-energy X-ray absorptiometry [18]. All blood samples were collected in the morning (8 a.m. to 9 a.m.) from the antecubital vein after a 12-hour overnight fast. All laboratory measurements were performed at the Institute of Clinical Chemistry and Pathobiochemistry, OvGU, Magdeburg, Germany. Fasting blood glucose, triglycerides, ALAT, ASAT, low-density lipoprotein (LDL) cholesterol, HDL cholesterol, and total cholesterol were analyzed by commercial enzymatic methods using a random-access analyzer (Modular, Roche Diagnostics, Mannheim, Germany). Lipoprotein fractions were analyzed by ultracentrifugation. Glucose was determined in sodium fluoride plasma. HbA1c was determined by high-performance liquid chromatography. Insulin was determined by 125I-radioimmunoassay (RIA) according to the manufacturer’s instructions (INSULIN-CT, CIS bio, Berlin, Germany).

### 4.4. Statistical Analysis

Data are given as median and interquartile range (IQR). Differences between independent samples (intervention vs. control at baseline or 6 months) were analyzed by Mann–Whitney U test. Paired samples were analyzed by Wilcoxon signed-rank test. Correlations between relative changes in parameters before and after the trial were assessed by Spearman’s rank correlation.

Calculations were performed using IBM SPSS Statistics, version 22.0 (IBM Corporation, Armonk, NY, USA). Results were considered significant at *p* < 0.05. The data analysis was performed using GraphPad Prism, version 8.0, R version 4.0.3., and RStudio version 1.3.959.

In order to identify which variables may influence osteocalcin metabolism, a Bayesian multiple linear regression was performed. Based on the low sample size, we decided to use Bayesian multiple regression analysis, which is a robust model. The posterior distribution of the linear regression was approximated using Markov chain Monte Carlo using the rethinking R package. The input variables were the ratio of relative changes (log ratio) between parameters before and after the trial period. The input variables were those that were significantly correlated to osteocalcin in the study (Figure 4, Supplementary Figure S1): ‘BMI-change’, ‘Leptin-change’, ‘Total Muscle Mass-change’, ‘ALAT-change’, ‘CRP-change’, ‘Insulin-change’, ‘TG-change’, ‘GFR-change’, and ‘Total fat mass-change’. To avoid multicollinearity, ‘Leptin-change’ was removed, since it was highly associated to insulin and glucose (not shown). Similarly, ‘Total fat mass’ was also removed, since it is highly associated with BMI (not shown). Both control and intervention groups were used as input. The likelihood of ‘osteocalcin-change’ follows a normal distribution, with the mean ‘osteocalcin-change’ and a standard deviation. The linear model is: osteocalcin ~N(α + ∑β_i_ x_i_,σ), with parameters being: α, the intercept of the regression, which represents the change in OC when all other parameters have changes equal to zero; β_i_, the slope of each variable; x_i_, which represents the rate of change in the dependent variable, “osteocalcin-change”, when the independent parameter changes by 1 unit. In addition, the following priors were used for the calculations: α~N(0,0.5), β~N(0,0.4), and σ~Expo(1). The hyperparameters were chosen to conform to a vague prior.

## Data Availability

Not applicable.

## Supplementary Materials

The following are available online at https://www.mdpi.com/article/10.3390/metabo11080526/s1, Figure S1: Correlation between OC, metabolic markers, body composition, and inflammation during the 6-month intervention in both groups, Table S1: Clinical parameters of participants in the control and intervention groups before and after the study period. Data are presented as median (interquartile range).
