# Supplementary material for: Osteocalcin Is Independently Associated with C-Reactive Protein during Lifestyle-Induced Weight Loss in Metabolic Syndrome
Source: Metabolites. 2021 Aug 9;11(8):526. doi: 10.3390/metabo11080526 (PMC8400285; doi:10.3390/metabo11080526)
Supplement: Supplementary file 1 [file metabolites-11-00526-s001.zip › metabolites-1268701-supplementary.pdf]

Table S1. Clinical parameters of participants in the control and intervention groups before and after the study period. Data are presented as median (interquartile range). The Wilcoxon signed-rank test was used to analyze differences between paired samples with n = 33 intervention, n = 30 control, * $p < 0.05$, ** $p < 0.01$, *** $p < 0.001$. No differences were found between treatment group vs. control group at baseline, analyzed by Mann–Whitney U test. Some parameters (indicated by [1]) were published in a previous study [18]. BMI, body mass index; LDL, low-density lipoprotein; HDL, high-density lipoprotein; TG, triglycerides; HbA1c, hemoglobin A1c; CRP, C-reactive protein; IL6, interleukin 6; ALAT, alanine aminotransferase; 25OHVD, 25-hydroxyvitamin D; GFR, glomerular filtration rate.

	Control Group		Intervention Group	
	0 Month	6 Month	0 Month	6 Month
**Age (years)** [1]	48 (43,75–51,25)		48 (45–51,5) n.s.	
**BMI** [1]	33,4 (31,7–37,1)	33,6 (32,4–35,8) n.s.	33,0 (31,2–35,8)	29,0 (27,3–31,4) ***
**Waist circumference (cm)**	114 (111–126,25)	114 (105,5–123) n.s.	116 (107,5–123,5)	105 (97–110) ***
**LDL cholesterol (mmol/L)** [1]	3,4 (3,0–4,4)	3,2 (2,7–4,1) n.s.	3,7 (3,1–4,7)	3,4 (2,4–4,1) **
**HDL cholesterol (mmol/L)** [1]	1,3 (1,1–1,5)	1,4 (1,1–1,5) n.s.	1,2 (1,0–1,4)	1,4 (1,2–1,5) ***
**TG (mmol/L)** [1]	2,0 (1,5–2,6)	2,0 (1,5–3,6) n.s.	2,1 (1,4–3,9)	1,4 (1,1–2,0) ***
**HbA1c (%)**	5,6 (5,3–5,9)	5,6 (5,3–5,8) n.s.	5,6 (5,3–6,05)	5,4 (5,2–5,6) ***
**Insulin (pmol/L)** [1]	62,5 (50–88,5)	61 (47–90,25) n.s.	88 (59–134,5)	47 (25–70) ***
**Leptin (ng/mL)**	10,9 (8,3–20,2)	13,4 (8,1–18,3) n.s.	12,5 (8,2–17,0)	5,6 (3,0–8,4) ***
**CRP (mg/L)**	3,0 (1,3–5,5)	2,5 (0,8–4,6) n.s.	3,0 (1,6–5,9)	1,1 (0,6–2,3) ***
**IL6 (pg/mL)**	2,1 (1,8–3,0)	2,5 (1,7–3,5) n.s.	2,6 (1,9–4,0)	2,0 (1,6–2,4) *
**ALAT (μmol/s*L)**	0,7 (0,5–1,1)	0,7 (0,5–1,1) n.s.	0,6 (0,5–1,0)	0,4 (0,3–0,5) ***
**25OHVD (ng/mL)**	22,3 (17,3–28,8)	23,6 (21,2–27,7) n.s.	22,4 (17,4–30,4)	24,2 (20–32,9) n.s.
**GFR (mL/min/1.73m²)**	95,0 (83,75–102,25)	89,5 (80,0–98,25) *	100,0 (93,0–106,0)	97,0 (92,5–104,5) n.s.

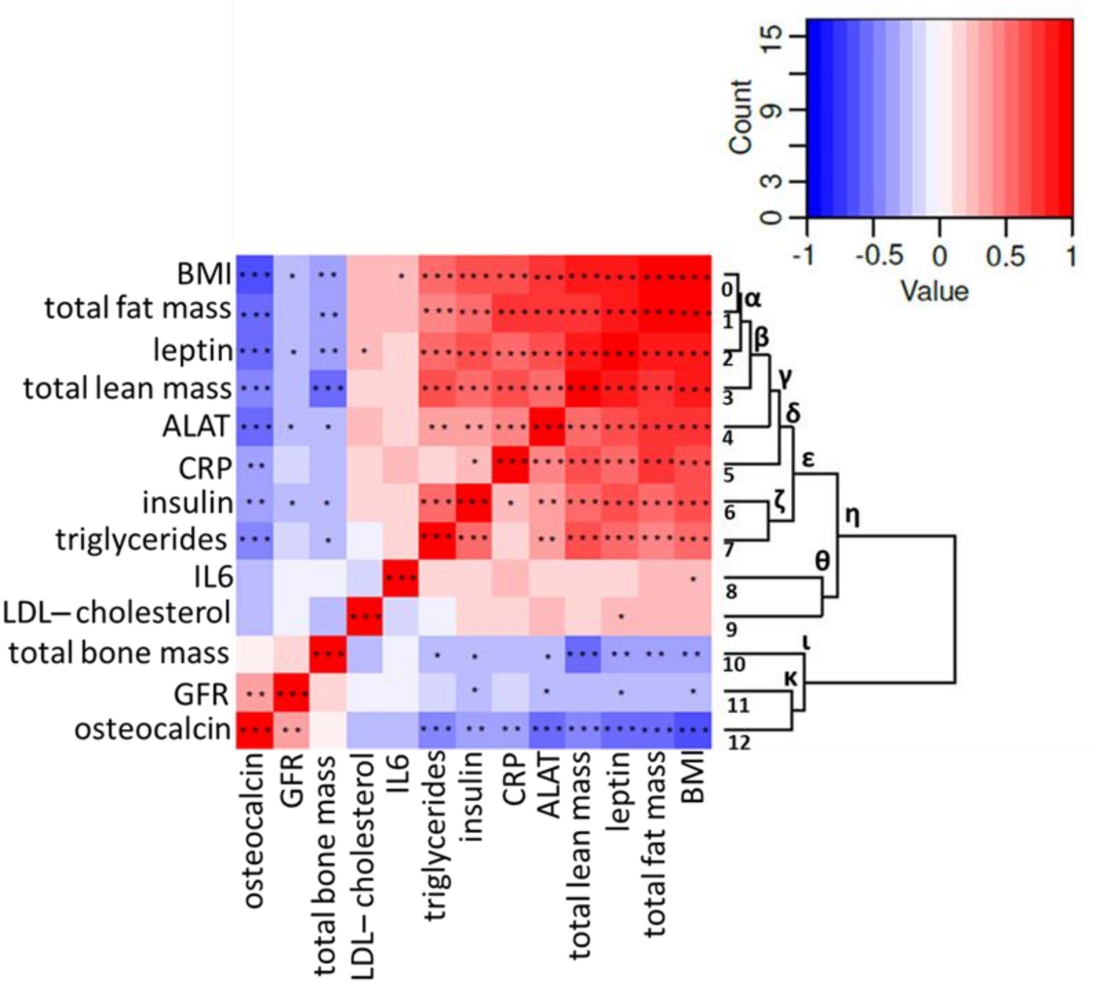

Figure S1. Correlation between OC, metabolic markers, body composition, and inflammation during the 6-month intervention in both groups. The dendrogram is a branching diagram that represents the relationships of similarity among a group of entities. Each branch is called a clade (α–κ). The terminal end of each clade is called a leaf. Clades can have just

one leaf (these are called simplicifolious) or they can have more than one. Two-leaved clades are bifolious. The arrangement of the clades explains which leaves (parameters) are most similar to each other. The height of the branch points indicates how similar or different they are from each other: the greater the height, the greater the difference. The term chunk represents each segment of the dendrogram at the parameter level, here 0–12. CRP, C-reactive protein; ALAT, alanine aminotransferase; BMI, body mass index; IL6, interleukin 6; LDL, low-density lipoprotein; GFR, glomerular filtration rate.
